# Patient perspectives on the causes of breast cancer: a qualitative study on the relationship between stress, trauma, and breast cancer development

**DOI:** 10.1080/17482631.2021.1983949

**Published:** 2021-10-25

**Authors:** Erica Niebauer, Nina Fry, Lisa A. Auster-Gussman, Helané Wahbeh

**Affiliations:** a Independent Researcher, Glasgow, Scotland, UK; b Research Department, Institute of Noetic Sciences, Petaluma, CA, USA; c Feinberg School of Medicine, Northwestern University, Chicago, IL, USA

**Keywords:** Breast cancer, trauma, adverse childhood experiences, stress, causal attribution

## Abstract

**Purpose**: We qualitatively evaluated breast cancer survivors’ perception of the relation between breast cancer development and both childhood trauma and stressful life events in adulthood.

**Methods**: Women (N = 50) who have or had a positive breast cancer diagnosis completed a close-ended survey, a timeline of significant life events, and an in-depth interview. All interviews were transcribed and inductively coded using thematic analysis with an emphasis on patient perspectives of illness.

**Results**: Participants reported a perceived connection between breast cancer development and stressful life events, and four themes were identified: 1) experiencing major interpersonal stress in both childhood and adulthood, 2) ideas about the relationship between emotional stress and physical disease, 3) ideas about how different types of stress contribute to developing breast cancer, 4) post-treatment post-traumatic growth and meaning-making.

**Conclusions**: Our findings suggest that of the participants who felt something could be causally attributed to their developing breast cancer, most of them made causal attributions between social, personal, and physical stress and trauma across the lifetime to the aetiology of their breast cancer. We suggest that breast cancer patients and survivors may benefit from additional psycho-social, stress-reducing, and/or somatic-based trauma-informed therapies to address stress and trauma.

## Introduction

Breast Cancer is the most common cancer diagnosis in women worldwide (World Health Organization, 2021). The World Health Organization estimated that 2.3 million women were diagnosed with, and 685,000 women died from breast cancer in 2020 (World Health Organization, 2021). These statistics suggest the ever-increasing importance of understanding survivors’ perceptions of their disease as breast cancer has numerous effects on individuals’ quality of life.

Patient perspectives of the relation between stress and the development of breast cancer have been previously examined. In two qualitative studies, 58.1% and 42.2% of women who cited a specific cause of their breast cancer development reported stress as this cause (Panjari et al., 2012; Stewart et al., 2001). Additionally, results from a systematic review of causal attributions of breast cancer by survivors reported 16 studies with evidence that survivors attributed their breast cancer diagnosis to stress, among other factors such as family history, environment, fate, or chance (Dumalaon-Canaria et al., 2014). A study on causal attribution by 299 breast cancer patients found that stress, diet and exercise were reported to be the most likely factors, while another study on 303 women who had survived breast cancer found that they attributed their breast cancer mostly to “stress and worry”, followed by “diet or eating habits” (Lee et al., 2021; Park et al., 2021). Another study of 159 women found that psychological causes were believed to be the reason why they developed breast cancer, while a further study identified that 46.4% of the women with breast cancer who were interviewed attribute their diagnosis to emotional or mental factors, especially stress (Peuker et al., 2016; Thomson et al., 2014). Another study on survivors of breast cancer reported that participants believed stress, followed by genetics and diet, were the most commonly attributed reasons for developing breast cancer, when they felt there was a cause (Kadhel et al., 2018).

While stress is not considered to be an official risk factor for developing breast cancer, the relationships between psychological, behavioural, and emotional events and breast cancer have been investigated, particularly regarding stress, trauma, and adverse life experiences. Results from a recent systematic review suggest evidence of links between stress, adverse life events, and the risk of developing breast cancer (Chiriac et al., 2018). Researchers have further suggested that stress has a “clear connection” physiologically to breast cancer (Antonova et al., 2011). For example, women who reported more adverse childhood experiences (ACES), such as emotional, sexual, or physical abuse or approximation to mental illness or addiction, had higher odds of a cancer diagnosis. However, this study was not limited to breast cancer expressly (H. Alcalá et al., 2017). Adverse life experiences and associated stress have also been related to disease progression and symptoms. For example, the experience of childhood emotional abuse is related to more intrusive breast cancer symptoms (Goldsmith et al., 2010), whereas women without stress or trauma history have been shown to have slower disease progression than those with a history of one or more incidents (Palesh et al., 2007). Moreover, childhood maltreatment is related to psychological distress symptoms and lower quality of life among women undergoing breast cancer treatment (Fagundes et al., 2012; Han et al., 2016; Janusek et al., 2012; Kuhlman et al., 2017).

This study builds upon previous literature evaluating cancer survivors’ general perceptions of breast cancer aetiology and extends it by examining more specifically breast cancer survivors’ perceptions of the relationship between lifetime traumatic events and breast cancer development. Specifically, we investigated whether survivors perceive trauma or stress as related to breast cancer development. The study’s research question was, “Do survivors perceive lifetime traumatic events, including physical, emotional, and energetic trauma (of a controlling, neglectful, or abusive nature), as connected to their breast cancer development?” We hypothesized that individuals might perceive the triggering of childhood trauma in adulthood as precipitating their breast cancer diagnosis. To investigate this hypothesis, we conducted an exploratory qualitative research study to evaluate survivors’ perceptions of the relationship between lifetime trauma and breast cancer development.

## Methods

### Participants

We recruited participants through the Institute of Noetic Sciences (IONS) listserv, blogs, general eNewsletter (membership ~65,000), Community Groups-specific eNewsletter, targeted email outreach, through IONS associated social networks (~85,000 followers), as well as our affiliate organizations’ social networks, women’s cancer centres, and social media pages. Fifty American women who had or currently have breast cancer were recruited to participate in the study. Study activities were approved by the Institute of Noetic Sciences Institutional Review Board (IRB) WAHH_2018_04. All participants met the following inclusion criteria: women, ages of 35–90, breast cancer diagnosis (all other cancers were exclusions), and currently accessing (or had regular access to) mental health resources at the time of the study.

### Procedure

Women who completed the screening and were eligible for the study were contacted by a research coordinator and provided with an online written Informed Consent. Consenting women completed an online questionnaire, a health and lifetime events timeline, and participated in a 1-hour phone interview. Participants who completed all three study activities received $150 for their participation in the form of a Visa Gift Card.

### Materials

#### Online survey

Participants completed an online survey through the SurveyMonkey platform (surveymonkey.com), which allows for HIPAA compliant data collection and provides detailed tracking of invitations and survey completion. Surveys were collected between 3 May 2019, to 17 July 2019. The survey included demographic information, general health, lifestyle questions, and breast cancer history (Table I).

**Table I.** Demographics, lifestyle variables, and breast cancer history

Factor	Level	Value [% or Mean (SD)]
Age, mean (SD)		55.9 (10.8)
Race	Non-Hispanic White/Caucasian Non-Hispanic African American/Black Hispanic/Latina/Latino Multiple Race/Ethnicities	90 2 2 4
In a relationship	Yes	47
Income	< $75,000 $75,000-$149,999 > $150,000	59 28 14
Number in household		2.4 (1.3)
Setting	Rural Suburban Urban	18 53 29
BMI		27.5 (7.1)
Exercise	Once per week 2 to 4 days per week 5 to 7 days per week I don’t regularly exercise	14 39 22 24
Age at first menses		12.7 (1.6)
Age at first childbirth		29.2 (6.9)
Moth took DES	No	93
Other chronic disease	Yes	29
Breast Cancer History		
Time since diagnosis (months)		71.8 (86.4)
Age at diagnosis		50.0 (10.6)
Method of first diagnosis	Manual exam Mammogram Ultrasound	41 31 57
Highest Diagnosed Stage	Don’t know/not sure Stage 0 (in situ) Stage I Stage II Stage III Stage IV	6 8 27 29 20 10

#### Timeline

Each participant submitted a timeline of their life’s most important moments and any breast cancer-related events via the SurveyMonkey platform or email (See Supplemental Data A for timeline instructions).

#### Interview

Each participant completed a 45–60 minute structured interview conducted by one of three researchers trained in interview and qualitative research methods. Probing questions were designed to elicit conversation about the overall research question and the timeline. To this end, the interview included questions on the following topics: 1) the event they believe impacted their life the most 2) events they believe reduced their vitality (or feeling “strong, vibrant, energized and positively engaged in life”) 3) any perceived connections between their childhood and adult experiences 4) events that they believe are associated with their developing breast cancer 5) experiences that increased or diminished a sense of “feminine nature” 6) their relationship with their mother or mother figure, 7) feelings of helplessness, hopelessness, anger or rage, and 8) any experiences with post-traumatic growth (See Supplemental Data B for full interview). The interview questions also referred to the participants’ timeline.

The interviews were conducted and recorded via Free Teleconference and subsequently transcribed and uploaded to Dedoose, a qualitative analysis program (version 8.3.17, Dedoose, Inc, Hermosa Beach, Ca). The recordings and transcriptions were de-identified before they were stored.

### Data processing and analysis

One researcher (EN) analysed the data using qualitative thematic analysis consisting of six steps (Braun & Clarke, 2006): familiarization with the data, coding, generating themes, reviewing themes, defining and naming themes, and reporting. As this was an exploratory study, the data were coded inductively using thematic analysis (Braun & Clarke, 2006). Themes were developed across the entire dataset rather than within individual questions. Quotations from the data are included to illustrate each theme and were edited with articles, punctuation, and extra clarification where needed to support the reading flow and comprehension.

### Participants and recruitment

Of the 237 women who began the survey, 187 were excluded for the following reasons: less than 34 years old (12), no breast cancer diagnosis (16), other cancer diagnoses (24), no mental health care (116), did not complete the survey (19), resulting in a final sample size of 50 women, all of whom did each of the three parts of the study. Percentages, means, and standard deviations of demographic variables, lifestyle factors, and breast cancer parameters are reported in Table I.

## Results

Four major themes were identified: 1) Experiencing major interpersonal stress in childhood and adulthood (childhood trauma, adult interpersonal stress), 2) ideas about the relationship between emotional stress and physical disease (stress builds up and causes the disease to emerge, stress makes the body vulnerable), 3) ideas about how different types of stress contribute to developing breast cancer (cumulative stress throughout the lifetime, relationship problems, distressing emotional experiences) and 4) post-treatment post-traumatic growth and meaning-making (“new life,” sense of community). The themes are reflected in Table II. Representative quotes for each theme are below.

**Table II.** A summary of thematic analysis themes and sub-themes

Themes and Sub-Themes			
Main Theme	Sub-Theme I	Sub-Theme II	Sub-Theme III
1. Experiencing major interpersonal conflict in childhood and adulthood	Childhood trauma	Adult interpersonal stress	
3. Ideas about the relationship between emotional stress and physical disease	Stress builds up and causes disease	Stress makes the body vulnerable	
4. Ideas about how different types of stress contribute to developing breast cancer	Cumulative stress throughout the lifetime	Relationship problems	Distressing Emotional experiences
5. Post-treatment post-traumatic growth and meaning-making	“New life”	Sense of community	

### Experiencing major interpersonal conflict in both childhood and adulthood

Most of the participants spoke of experiencing recurring interpersonal conflict across their lifetimes. Stressful or traumatic events in childhood and relational conflict in adulthood were experienced in varying degrees by nearly all the participants. Nearly every participant described adverse events that occurred in childhood as significantly impactful to them. Various types of relational stress in childhood were discussed, from more mild stressful events such as a general feeling of low self-esteem or feeling “left out” to more extreme stressful events such as bullying, emotional, verbal, sexual, or physical abuse. In adulthood, nearly all the participants described the recurring stressful interpersonal conflict with others, primarily concerning romantic, familial, and professional relationships, significantly impacting their lives.

#### Childhood trauma

The majority of participants recalled a variety of events in their childhood that they considered to be stressful. These events involved parents, family acquaintances, and peers. They included relational issues such as bullying or abuse and uncomfortable feelings of confusion, and low self-esteem. For example, one participant described how they felt their parents mistreated them as a child:

… I think it was just like … a series of events, or … the way my parents were raised was the way that they raised us, which wasn’t perfect … a lot of sort of verbally abusive comments, and some bullying …

Another person described a feeling of low self-worth as a child:

… Although I don’t remember experiences about a trauma as a kid, with the reflection and therapy and counseling that I’ve had throughout my lifetime I really think that sort of like … neglect … had the biggest impact of all those things … for most of my life I felt like it wasn’t in a real conscious way … like I had a low perception of my worth … And that has been challenging … I know it just thinking that I’m not that valuable. Sort of (on) a core, subconscious level.

Another participant described the experience of sexual assault, which significantly impacted their life:

… I would say the biggest impact were the assaults that happened to me at age, I think I was 13. I might have been 12, but in any event, it was in junior high. That I think was huge, more than I ever realized …

Another person remembered feeling hopeless as a child due to being bullied at school:

… I remember, especially in junior high … I just felt very hopeless about going to school every day. I knew I was just going to get teased and tormented and feel really uncomfortable. I know I’ve had a lot of moments where I’ve just felt hopeless about making friends.

#### Adult interpersonal stress

In addition to childhood stress, participants frequently mentioned experiencing stress in adulthood. The majority of this conflict was interpersonal and stressful, and included family, romantic partners, friends, children, and co-workers. One participant felt that issues with her ex-husband and children were particularly stressful:

… My children totally cut me off because they blamed me for being responsible for the divorce, and that was fueled very much by (my ex-husband’s) new wife who played on that as much as she could. So, I lost my communication with the two most important … people in my life, my children. And, you know, you think of your breast as being the source of their nourishment when they’re babies. And that’s why I think it affected me there.

Another participant explained how feeling disconnected from her daughter was traumatic for her:

Well, I guess just from a trauma standpoint, I do EMDR now, a type of therapy, and she says we work with ‘little t’ trauma. It’s funny, I feel like I’m one of very few women that hasn’t been sexually abused, or raped or something, and obviously those are ‘huge t’ traumas … the divorce for me, I remember having this feeling of my daughter is getting pulled away from me …. And I felt like my heart was just getting ripped out of my body. I’ve never felt anything … like I couldn’t breathe, I couldn’t cry, I just felt like I was getting ripped inside out. And that happened for a long time … it was like … a couple of years … and we just fought about custody in court for a year and … I felt like I had died during that time.

Another person described how she felt helpless in her romantic relationships:

I mean when my son was first born, I felt like I didn’t know how to do anything. I don’t know if that was really a feeling of helplessness as much as just floundering … just being a new parent. What really sticks out though is I had this one relationship right after my first child was born …, and it was not a very good relationship. He wasn’t a good partner. And I felt like I had nowhere to turn. Like none of my support systems were there … I really was pretty isolated. And for a while, I felt very kind of trapped, and helpless in that relationship.

Most participants experienced significant interpersonal stress at some point in their lives, and they referred to such stress as significant life events. These conflict areas were often located within the family, in romantic relationships, or with co-workers or employers. Notably, many participants felt that these events contributed to their stress, leading to their breast cancer development.

### Ideas about the relationship between emotional stress and physical disease

The majority of participants identified a relationship between emotional stress and physical disease in the body. Specifically, the idea that emotional stress can cause physical illness alluded to the idea that there was a connection between personal life experiences and physical effects on the body. Stress was considered to relate specifically to participants’ own experience with breast cancer. However, there was also a broader concept that stress was related to developing the disease through various mechanisms, mainly through cumulative stress causing the disease to emerge and stress making the body weaker or more vulnerable to disease.

#### Stress builds up and causes disease to emerge

Many participants felt that stress and emotions could physically impact the body and potentially cause diseases to develop. Stress was conceptualized as something that could accumulate, both in the frequency of stressful situations and in its general presence in the body. Many participants thought that accumulating stressful events over time could cause disease to emerge from within them. One person made connections between cumulative stressful life events as triggering various health conditions (as she otherwise considered herself to lead a healthy lifestyle):

… it made me really think about the stress in your life and how it can impact some of your physical things. When I looked for example, at my ovarian cyst … that was right around the time I was going through the divorce. And then when I looked at my migraines, I thought … I got those … at the end of moving out of my house and it ended after my divorce … my thyroid condition at age 56 … that was right around the time I lost some people in my life. And I thought, wow, could some things have triggered … the stress in my life. Because I’m a very healthy person, I take really good care of myself so I always wondered why would I get breast cancer? And then I was like well you’ve been under stress almost your whole life … that timeline was very eye opening for me.

Another participant stated that cancer had “lived” in her body since she experienced the stress of her childhood, which is where it emerged from in adulthood as a result of accumulated life experiences:

… I think it’s a whole picture … your life experiences are your life experiences, and they add up, and what happens is what happens, and I think your health issues have something to do with that. And the other thing that I felt is that, what’s wrong, because I have a really good life now, I really do. And, you know, my bad childhood is just like, I felt it was more living in my body that I couldn’t get rid of it, rather than in my thinking mind. I understand, you know, it’s obviously both places, but I just felt like, oh, I need to do something to get rid of this, and it lived in me.

Another person described how emotional and mental stress that had built up made her cancer worse over time:

My cancer was probably in process for many years but the mental stress and everything that I was going through the last few years is what made it so on fire. It wasn’t about the physicality, it was about what was going on inside me mentally, emotionally, it really made it happen that way.

Finally, another participant felt that stressful events which accumulated over time “pushed” cancer in her body into developing further:

… what I have felt over the last three years I’d say is that I have been under such insane emotional stress, and verbal abuse from two boyfriends …. and because of the gut feeling that I had while I was being verbally abused, and what that made me feel like afterwards, and how things have triggered me since that I feel like there’s a connection … Even though I know that it’s probably likely that the cancer in my body was set up, you know, a little earlier than these particular events … one leads to another, these events were the qualifying events that pushed me over the edge, and into the zone of allowing … my body (to be) so … depleted, and so emotionally damaged, and psychically damaged … I don’t know that I’ll ever get over those things that I heard, but I definitely feel that those had an impact on push coming to shove … I’ve been religious, and having my mammograms for 30 years, and nothing has ever been seen outside of, you know, dense breast tissue. And then, this last year was the year that something was seen, and then … that’s where push became shove.

#### Stress makes the body vulnerable

Participants felt that stress could cause cancer to grow faster and make the body weaker, so it cannot “fight it off” as well. The concept of the body being vulnerable to cancer due to stress is related to the idea that stress breaks down the body’s defences, which is the point when cancer has a chance to emerge or “take hold.” One participant felt that the stress of a situation itself (being separated from her child) made her body more vulnerable, and this vulnerability made her body open to the breast cancer “taking over”:

I haven’t had anyone die who’s close to me. But I’ve definitely experienced a huge death of being separated from my child because I was a stay at home mom, breast fed her for tons of years and very, very connected to my kid and still am. … It was absolutely horrible. I’m surprised, like I do attribute a large part of (that to) getting cancer, but I want to say the vulnerability of my own body I think I became very vulnerable just due to that stress event, that anything could have occupied me and taken over, so I’m kind of not surprised. I think cancer is a combination of things, but I think that’s a huge contributing piece.

Another participant explained that emotional experiences such as trauma, stress, and anxiety are “hard on the body,” which makes one more susceptible to developing cance:r

I would say that traumatic events in your life lead to stress, and anxiety, and both stress and anxiety are very, very hard on your body, and so you’re almost allowing something like breast cancer to happen. I’m not saying you chose it … I didn’t choose that, but I think that anytime we go through a high level of trauma, or something very significant, it’s going to have an effect on you physically as well as mentally, and emotionally. And absolutely anytime that you do something that alters your body, you’re allowing … I don’t think I’m explaining this right … I’m making myself more susceptible to getting cancer … if I don’t eat right, if I don’t exercise, if I have trauma …

Another participant explained how stress caused her body to be less able to “fight off” cancer already present in her body:

… I think everything was building up. I mean I think on a physical level, I had just exhausted my body to such a great degree just from the stress that I think that maybe it could have fought off if there were … the little scout cells … if the cancer was just brewing. If I had been in a better physical condition, and not so stressed, I wonder if my body could have fought it off. But … I don’t know that my father dying … I wouldn’t attribute it that much to that, but I would say mostly - I would put it more towards the health.

Finally, one participant felt that the reason she got cancer is that stress caused her immune system to weaken and created a “terrain” for cancer to develop:

I would say the events of my life in that last couple of years, stressful marriage, moving to Europe, moving back home, then surgery, jaw surgery, the failed hysterectomy, all the stress and all the things from that, my feeling about it is that something that might have been low grade maybe would have never even turned into anything huge, went crazy. Like (it) grew from the stress and effects on my immune system. And all the exposure, the radioactive dyes. Put the whole package together (and) it created a terrain for something that might have been mild and almost fairly undetectable to … go crazy and produce five tumors in my breast in one year. Because I had a clean mammogram one year before my diagnosis. And then I have five tumors.

The specificity of the concept of stress “building up” in and “breaking down” the body was a recurring theme among participants. Participants conceptually viewed diseases such as cancer as something that “takes root” in the body or emerges and “takes over” as a result of stress accumulating. The view of the patient’s own body as a site of accumulated stress points to the highly personal aspects of the disease, as participants related their cancer to their own stressful emotional and social experiences throughout their lives. While participants perceived the experience of stress to have a substantial impact on their well-being, the participants also attributed a relationship between their emotional life experiences and outcomes of their physical health, specifically in feeling there was a pathway between stressful events and cancer.

### Ideas about how different types of stress contribute to developing breast cancer

Approximately half of the participants personally attributed a specific cause to their breast cancer development related to stressful emotional or social experiences. Participants who did attribute a specific cause to their developing breast cancer cited events such as relationship issues, general stress, negative emotions, mental health issues, a physical event, such as another illness or injury, or a culmination of stressful events over time. Of the other half of participants, some were uncertain of a specific event that may have contributed to their developing breast cancer but suggested possible relevant events, while some others said they thought no specific events contributed to their developing breast cancer.

#### Cumulative stress throughout the lifetime

Cumulative stress was often cited as a reason breast cancer may have developed. Instances of cumulative stress included interpersonal, career, and physical stress and featured over the lifetime from childhood to adulthood. One participant described how a series of stressful events happened for a few years before her diagnosis, which made her identify the connections between the two:

Well, I wrote a book, and I did it in six months. I had a co-author. But, it’s kind of unheard of to write a book that fast. That was a pretty stressful experience. And that happened – let’s see, we published in 2004, and I was diagnosed in 2006. So, you know, I had a stressful marriage. I had, you know, this book thing – I mean I do wonder about that. I just wonder whether that was – you know, I mean I was a newspaper reporter. I was used to being in a high-stress kind of situation, but I kind of wonder if at that particular time, that may have, you know, pushed my health over the edge in some way.

Another participant explained how stressful relational problems throughout her lifespan caused her to get “sick over time”:

… I was constantly having to … fight for myself (at work). A common … subtext throughout … most of my life is not being believed and having to … advocate on my behalf in a way that has been so hard on me that it caused me to get sick over time. Whereas, had I had the supportive environment at home, or supportive environment on the job, or the support of physicians who … understood what was going on with me, things would have been different, but they weren’t.

Another participant felt that an accumulation of stressful events coincided with her discovering a lump that turned out to be breast cance:r

I’ve often questioned if it was brought on because of my couple of years prior to finding the lump were quite stressful, the one dealing with my fiancé’s ex and having to deal with step-kids and then also my job was just increasingly demanding. I had a specific person at work that was going out of her way to make my life difficult. Ultimately, I quit the job because of how stressful it was. I could tell that it was not good for my health … I literally found the lump the day after I quit. So, for me I feel like it was like the universe going like this is not good for you. You’re gonna have a bigger hurdle to jump over. And the timing just felt eerie. It was like I quit at 11pm the night before and at 7am my fiancé found the lump. So, it wasn’t even a 24 hour turnaround when that happened. It was crazy, yeah.

#### Relationship problems

Relationship stress, mainly related to romantic relationships and issues within the family, was often cited as to why someone felt they developed breast cancer. Relational abuse, illnesses within the family, and “falling out” with people were examples of specific relationship problems that participants experienced. One participant described how the “toxicity” of her marriage caused her to get cancer:

I don’t know if there are any specific events. But, I do think for me, it’s related a lot to the toxicity of my relationship within my marriage. And there’s various events … throughout my marriage. I mean … arguments, or the way we related to each other, the way I was treated, things like that. It was like … the whole picture of my entire marriage that contributed to my breast cancer. So, it’s not one specific event. But … I do remember … when I was going through my cancer treatment … it was one of the chemo treatments … I was feeling nauseous, and I am on the bathroom floor, and I am shaking, and I am … hugging the toilet. And my husband comes and starts yelling at me … because my daughter was crying. And I remember thinking at that moment that … there’s something wrong here … the way he is yelling at me … this shouldn’t be. And I remember thinking that that was cancer. So … that moment was the first time I saw how … the situation I’m letting myself be in is causing me to get cancer. And it was that day I took off my ring.

Another participant explained how the stress of dealing with illness in her family contributed to her exhaustion, leading to her developing cancer:

I had a lot of stress in the couple of years before my cancer, with school, and my dad, and that kind of stuff. I’m not sure what else I would link with it …. especially when my dad was sick, I was with him five days a week, and then I would drive – he was about 100 miles away from here where he lived, and I would drive home on the weekends, and my sister would go take care of him. And I just felt like I wasn’t seeing my family enough, and I was always on the road, and it was very – physically, and emotionally exhausting.

Another person reported that arguments within her extended family contributed significantly to her stress, which may have “brought on” her breast cancer:

… A few years before being diagnosed with breast cancer I did kind of have a big falling out with some family members, especially my husband’s family. There was a lot of stress with that relationship. And it got to the point where I didn’t see them for a year. So sometimes yes that was a very upsetting time for me. And I myself sort of maybe, it was very, very stressful, that might have brought things (breast cancer) on … there’s always been issues with the family … I would say at that point a couple years before my breast cancer I did feel quite traumatized by it.

#### Distressing emotional experiences

Many participants also felt that psychological or emotional events, such as depression, hopelessness, being overwhelmed, or experiencing mental illness, may have somehow been related to their developing breast cancer. One participant did not feel certain that any one event caused her breast cancer but made the connection that her diagnosis was associated with the negative experience of her depression:

I don’t know. Let me clarify. I do think my breast cancer is genetic and environmental, with a strong psychological component. And I don’t know what that component is … I don’t know if it’s hopelessness. I don’t feel like it’s the depression. I think it’s a component of the depression. I think it’s that hopelessness. And not seeing a way out of something …. I never actively wanted to kill myself, but it was like, what am I living for? I think if anything that’s the theme that I associate the breast cancer with. But I can’t connect it to one event or say, oh, I got this because my marriage or I got this because my father …

Another participant explained how feeling overwhelmed with her life led up to her diagnosis:

I was feeling kind of – I mean sort of like the main stage things were pretty, my work was good, my daughter doing great, my relationship was difficult, but I had a nice – my home environment otherwise was nice. I had a lot of friends and support. But I was feeling like I just wanted to slow everything down. I felt like life was just too fast and there’s too much happening. And I remember telling my friend, I just want to stop working. I just want to not work for a while. And I really thought about that in retrospect. That was about five, six months before I was diagnosed.

Another participant felt her period of depression before her diagnosis was to blame for her developing breast cancer:

… Well, the year leading up to it, I was feeling so depressed that I would think about never killing myself, but, you know, if a car were to hit me, that might be – welcome [laughter]. This was really a dark time …. And … my mom had had breast cancer … stage one when they found it … it was a whole different kind of thing than I had. But, I always expected to have it, but I also always expected to survive it too. So, I wasn’t real surprised when I was diagnosed. And I’ve always blamed it in a way on that period of time when I was so miserable. Actually, having the breast cancer changed my life, and I’m never sad anymore.

The majority of the participants believed that their breast cancer was caused, in part, by stress or traumatic or interpersonal issues across the lifespan. Participants perceived a relationship between stress, particularly of a social or emotional nature, and breast cancer development, which suggests that patients may situate their experience with breast cancer within the larger narrative framework of their lives as a way to understand their diagnosis. Since breast cancer emerges in adulthood and is usually an emotionally challenging experience, connections between personal events and a diagnosis potentially allow patients to draw on their personal, embodied experiences of the disease to ask existential questions such as “why me?” when thinking about their breast cancer experience and the reasons why they may have developed breast cancer.

### Post-treatment post-traumatic growth and meaning-making

Most of the participants felt that having breast cancer led them to experience post-traumatic growth. People identified connections between their experience with breast cancer and self-improvement, improving their relationships with others and giving them a larger sense of belonging to a community of other survivors, their medical team, and their local communities. Many participants also felt that breast cancer allowed them to re-evaluate what was important to them in their lives and allowed them to start a “new life.” Additionally, many participants felt that participating in the research brought them a sense of contributing to something larger than themselves by potentially helping others.

#### “New life”

Many participants felt that having breast cancer was a learning experience for them and allowed them to change their lives. One participant described how she felt that cancer gave her a new appreciation for life:

… I think there was definitely a silver lining to the cancer …, like most people that helped me appreciate life more knowing that … life could be taken away at any moment. I was kind of an overachiever before I got the cancer. So, I was working a full-time job … So, having the cancer … the universe is saying, “You need to slow down.” So, I got out of politics. And then, I had the time to start dating again. And that’s when I met my husband … I feel like that was a really good positive thing to happen. I think that most major stressors … once it’s over, I feel better about having … survived it, and maybe learned some lessons.

Another participant felt that she was able to move forward with her life quickly after her experience with breast cancer:

Well, because of all this, I moved out here … where my life has been extremely happy because of my involvement with volunteer activities … And those have all been happy, confirming experiences that I look back with such joy, and such pleasure, so much delight that I am doing those things. And I wouldn’t be if I was still back (there) in a sense, mourning because of, you know, being in familiar places with negative memories. So, I was spurred to come out here, because that was my chance to get away, and start a new life.

#### Sense of community

Many participants felt that having breast cancer gave them a sense of connecting to and helping others, leading to feeling the benefits of prosocial behaviour. One participant discussed how supporting others with a similar diagnosis helped her process her own:

Yeah, I did something actually through … the hospital. They had something where they would connect you … and you would help support other people going through a similar diagnosis of what you had. So, you could share your experience, and be there to support them while they went through it. And I did that for a while too. So, all of those things helped me process what I was going through, but then also allowed me to help others.

Another participant explained how her experience with cancer connected her to people she did not expect:

In some senses it’s helped me to just see a sense of beauty within darkness. And with cancer it’s interesting because it seems like in some ways it brings people together. There’s a lot of people I’ve connected with and made friends with who I probably would never have connected with if I didn’t have cancer. And just the outpouring of support and love that people have given me throughout my process has been amazing.

Another participant felt that her diagnosis “opened doors” to a new community of people:

The breast cancer, what it did for me, it opened – it sounds terrible, I don’t mean it that way, but it opened so many doors of, I would say like compassionate, a community – just the whole community surrounding breast cancer, other women who have experienced it, the support teams, and people that help you get through it, the doors that opened, and the opportunities that opened to do volunteer work, and to work as an advocate were huge …

Post-traumatic growth has been reported among breast cancer survivors, suggesting positive attitude changes towards oneself and life after treatment (Barthakur et al., 2016; Paredes & Pereira, 2018). The majority of participants reported that they had experienced post-traumatic growth due to their breast cancer experience. Breast cancer’s impact on participants through positively changing their worldviews implies that despite the idea that interpersonal stress or trauma may have contributed to their diagnosis, their breast cancer experience ultimately helped redefine their relationships and allows for more profound interpersonal connections. This meaning-making is valuable because it potentially helps transform the participants’ experience of the disease from interpersonally painful to positively interconnected and ultimately pro-social.

## Discussion

Although cancer aetiology is examined from various approaches and perspectives, this study’s purpose was to examine patient perspectives of the determinants of their cancer development and progression through qualitative, in-depth interviews. Investigating the perceived causes of breast cancer from a patient’s subjective standpoint is useful because it situates the phenomenon of breast cancer within the patient’s experience. Patient narratives, particularly regarding why participants thought they developed breast cancer, can be useful to understanding the experience of the disease itself. According to Nordenfelt’s philosophy on “The Reverse Theory of Disease and Illness,” a patient’s subjective experience of having a cluster of symptoms not only contributes to further understanding and treatment of a specific illness but is also the necessary precursor to the identification of oneself as having a disease (Nordenfelt, 2007). Our aim in this study was to use this theoretical framework as a starting point to examine ideas about breast cancer. From this perspective, where the patient’s experience and self-beliefs are expressly valued, we find that many breast cancer patients perceive that their stress and trauma experiences and lifetime stress contributed to their breast cancer development and course, making stress a significant factor in the experience of having breast cancer itself. Specifically, a majority of participants did, in some regard, believe that various emotional and interpersonal factors contributed to breast cancer development above and beyond environmental or genetic factors, and many felt they were able to pinpoint a specific stressful cause for their illness.

While the majority of participants reported experiencing some level of childhood trauma or interpersonal stress across the lifetime, our hypothesis that individuals might perceive the triggering of childhood trauma in adulthood as precipitating their breast cancer diagnosis was generally not held by participants. However, of the participants who did attribute a cause to their developing breast cancer, the majority causally attributed trauma and stress to their breast cancer diagnoses, specifically through the effect of emotional stress on the physical body itself. Many participants believed that the accumulative effect of stress, as well as the “weakening” of the body due to stress, may lead to the emergence of breast cancer. While diagnosis and treatment of breast cancer may itself be traumatic or stressful, our research shows that patients’ trauma and stress during their experience with breast cancer can also be related to more personal and existential experiences, particularly due to early childhood trauma or interpersonal stress. Our results find that many women who have or have had breast cancer often attribute their developing breast cancer to trauma and stress which occurred across the lifetime due to the impact of these specific types of stress on the body. While not scientifically proven, these causal attributions by the participants point to the presence of stress while receiving treatment for breast cancer, which may inform the usage of further emotion-based therapies for patients undergoing treatment for breast cancer.

Additionally, studies show that participating in stress-reducing treatments and therapy while undergoing breast cancer treatment, while not improving survival rates, can improve both quality of life and adjustment. One RCT showed that supportive-expressive group therapy reduced trauma symptoms, prevented depression, reduced “hopelessness-helplessness”, and improved social functioning (Kissane et al., 2007). Another 10 year follow-up study looking at the effects on an adjunctive psychosocial support program for breast cancer patients showed that while survival rates did not increase, quality of life did (Gellert et al., 1993). Another study on incorporating relaxation training as a part of treatment for breast cancer patients improved psychological distress during hospitalization (Kovacic & Kovacic, 2011). A systematic review and meta-analysis on mindfulness based stress reduction improved psychological distress in breast cancer patients, while another study on individual psycho-social support for breast cancer patients reported better psychological outcomes (Arving et al., 2007; Cramer et al., 2012). Additionally, a study by Dumalaon et al. found that attributing stress to the development of breast cancer was “significantly associated with lower psychological well-being” (Dumalaon‐Canaria et al., 2018). Due to the impact of attributing stress to their developing breast cancer, as well as the impact of stress across the lifetime in general, therapeutic interventions specifically regarding stress and psychological issues may be of great benefit to breast cancer patients undergoing treatment.

These findings are useful in that they can provide information regarding how women with breast cancer experience their diagnosis and treatment in order to support the benefits of additional psycho-social, stress-reducing, or trauma-informed therapies during and post-treatment. Because women felt that breast cancer may be a result of trauma or stress, and they also felt that having breast cancer was a transformative experience for them which stimulated post-traumatic growth, it might be beneficial for therapy providers to frame the breast cancer experience as both a means of physical healing as well as an emotionally therapeutic process through which early childhood trauma or current interpersonal stressors can be addressed through psychological support, potentially improving health-related quality of life and adjustment. Knowing about these ideas held by women undergoing breast cancer treatment allows us a deeper and more holistic insight into the potential benefits of supplemental emotional care. Therefore, we recommend providing psycho-social, trauma informed, and/or stress-reducing support for breast cancer patients to alleviate stress associated with childhood trauma and interpersonal stress during treatment. Additionally, because many women felt that stress has a substantive effect on the physical body itself, it may be of benefit for such women to utilize somatic based emotional therapies.

Finally, womens’ experiences, particularly regarding influences of stressful, emotional, and interpersonal experiences relating to breast cancer, warrants more research and investigation. It is essential to seek a deeper understanding of the potential relationships between traumatic life experiences, interpersonal conflict, stress, and breast cancer and incorporate traumatic histories and patient narratives into psychosocial treatment areas for breast cancer patients. Further research should look deeper into the influence of ACES and early childhood stressful experiences on breast cancer, but should also look into stress occurring across the lifetime. Particular attention should be paid to adult relationships and long-term emotional and interpersonal issues and their potential impact on the breast cancer experience.

## Limitations

The main limitation of the study is that the specific questions being asked necessarily inform the answers given. Regardless, the results can inform future studies and therapeutic interventions.

## Conclusions

Participants frequently discussed how childhood trauma and adult interpersonal stress significantly impacts their lives and breast cancer experiences. There was an overall idea from the participants that these events may have contributed to their developing breast cancer, specifically regarding lifetime stress’ cumulative effect on the body or stress making the body more vulnerable in some way. Additionally, although childhood and adult interpersonal conflict were perceived as contributing to breast cancer development, entering remission appeared to promote feelings of increased social connection and post-traumatic growth. We recommend that providers working with breast cancer patients consider providing emotionally therapeutic resources, particularly with a somatic, psycho-social, trauma-informed or stress-reducing focus which address trauma, stress, and interpersonal issues, in order to help patients conceptualize their breast cancer treatment as an overall psychologically healing process, which may positively influence adjustment and quality of life.

## Supplementary Material

Supplemental Material
