# Supplementary material for: Patient perspectives on the causes of breast cancer: a qualitative study on the relationship between stress, trauma, and breast cancer development
Source: Int J Qual Stud Health Well-being. 2021 Oct 25;16(1):1983949. doi: 10.1080/17482631.2021.1983949 (PMC8547822; doi:10.1080/17482631.2021.1983949)
Supplement: Supplemental Material [file ZQHW_A_1983949_SM9962.zip › Supplementary files/Supplemental Data A Timeline Instructions_.docx]

**Life Experiences and Breast Cancer Interview**

**Timeline Instructions**

Thank you again for participating in this study! Below are the instructions for how to complete your timeline and how to submit it via email prior to your interview. This timeline must be completed and submitted to the Research Program Manager prior to the interview, or your interview will need to be rescheduled.

**Timeline Overview**

The purpose of this exercise is to give you an opportunity to record vital information about your lifetime experiences and your breast cancer health history on a timeline. This exercise should take about 15-30 minutes.

Below is an example of a completed timeline to give you a sense of what yours might look like when you have completed the process. *This example of a Health Map is not related to breast cancer but other health issues.* (While this example does not relate to breast cancer directly, please use this as a guide of how to track major life experiences and breast cancer experiences on your own timeline). Please read all the instructions below before beginning.


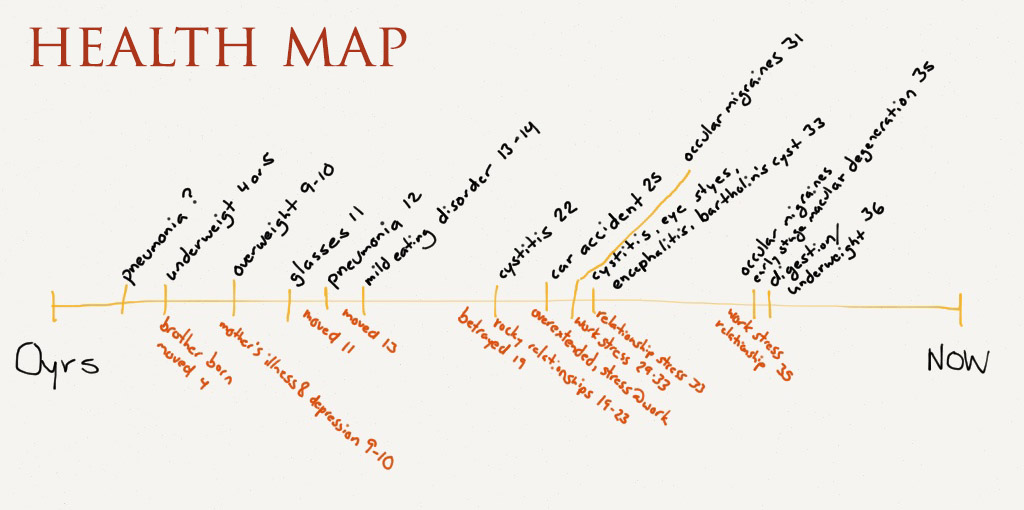


**Step 1: Preparation**

Gather the following items:

- Markers, pen or pencil
- Several sheets of paper (8x11 or larger)

**Step 2: Draw a Time/Life-Line**

Draw a line horizontally across the page and start with age 0 (when you were born) on the far left and your current age on the far right. You may want to create marks on the timeline for the time periods we ask about in this study (ages 0-7, 8-18, 19 - now).

**Step 3: Enter Lifetime and Health Events**

In this step, you will put your important life and health experiences in chronological order of your age on the timeline:

- Draw a short vertical line on your timeline for each of the key events that you feel impacted you (lifetime and health) – allowing ample space between events so that you can write in details.
  - As on the timeline example above, place your breast cancer health events ***above*** the timeline and your lifetime events ***below*** the timeline.
  - Allow ample space as recording one event can trigger a memory of another.
  - Remain open to adding events as you complete the timeline; it’s natural for one event to trigger a memory of another.

**Step 3: Submit to Research Time Prior to Your Interview**

Once you have completed your Timeline, please upload either a photo or a scan of your timeline and email to . In the subject line of this email please include the unique study ID that you were provided to complete the study. If you cannot remember your study ID, please contact at the email above or at for that information or any other questions.

This is a confidential email address and your timeline will not be seen by anyone other than the Project Manager, the interviewer and the Director of Research. Your timeline **must** be submitted prior to your interview.

Thank you so much for completing this Timeline Exercise! Please keep your timeline for when we complete our interview. We will be in touch with you shortly to schedule your interview.
