# Supplementary material for: Patient perspectives on the causes of breast cancer: a qualitative study on the relationship between stress, trauma, and breast cancer development
Source: Int J Qual Stud Health Well-being. 2021 Oct 25;16(1):1983949. doi: 10.1080/17482631.2021.1983949 (PMC8547822; doi:10.1080/17482631.2021.1983949)
Supplement: Supplemental Material [file ZQHW_A_1983949_SM9962.zip › Supplementary files/Supplemental Data B Life Experiences Breast Cancer_Interview Questions_6_21_19.docx]

# Life Experiences and Breast Cancer - Interview Questions

***Interview Script***

Thank you so much being part of this study. As you read in the consent form, we are doing these interviews to find out more about how your life events may be related to your breast cancer experience. Before we begin, please know that you can skip any question you don’t want to answer, take a break or stop the interview at any time, and there are no right or wrong answers to these questions. We are on an exploration to understand the relationships, ***if any,*** between events in our lives and health. For your participation, you will receive $75 of compensation in the form of a Visa Gift Card regardless of what your answers are on this interview, if you complete all three activities, the survey, timeline and the interview. Since you completed your questionnaire and timeline before June 21st, you will also receive an additional

$75 Visa Gift card.

Do you have any questions before we get started?

*Timeline Check-In*

Ok, great. I see that you have completed your timeline. Do you have that in front of you right now? *(if not, please take a moment to get your timeline so that you have it during the interview)*

You can reference your timeline as we go through our questions. You will also have time at the end to talk about any other events on your timeline that we don’t directly ask you about.

*Interview Questions*

Ok, here is the first question:

As you reflect on your timeline experiences, are there any events that you feel impacted your life the **most**?

- If yes, how old were you?
- Can you share more about the event, and how it affected you?
- *[If participant seems stressed in any way..”Are you okay if we move on to the next question, or do you need a break?”]*

1. About the events on your timeline again, are there any events that you feel reduced your vitality in some way? What we mean by vitality is feeling strong, vibrant, energized and positively engaged in your life. You can take a minute to think about that.
   - If yes, how old were you?
   - Can you tell me more about the event and how it affected you?

Thank you for sharing your experience. Is there another event on your timeline that you feel reduced your vitality? *Note to interviewer: Repeat Question #2 (sub-questions for Events 2 -3 - limit to 3 responses)*

Thank you for sharing about your experiences. *[If participant seems stressed in any way or the previous section was long and/or emotional…”Are you ok if we keep going, or would you like to take a break?”]*

1. In looking at your timeline, I see *[describe event - look at timeline for any events before diagnosis].* Can you tell me more about the event and how it affected you?

(*Note to Interviewer: This question is exactly the same as the one before but is trying to get at any events that happened right before their diagnosis. If they already talked about this, you wouldn’t ask this question. If they had one on the timeline that they didn’t talk about that was right before their diagnosis you would ask about it).*

Thank you for sharing about your timeline events.

1. I’m going to switch gears a bit here and ask about some connections others have reported and see if they are true for you, although they may not be, which is totally fine.
   - Some people feel that very difficult circumstances or traumatic events in their **adulthood** can unlock, trigger, or bring back traumatic experiences from **childhood**. Do you think this might be true for you? If so, can you explain more about how you experience this?
   - Some women who have experienced breast cancer associate some events in their life to their breast cancer in some way. Is this true for you? If so, can you share more about that?
2. Thank you so much for sharing about that potential connection. We are going to change topics again for the next few questions.
   - We all have a masculine and feminine aspects to us. For example, we all can be gentle and nurturing to children, which would typically be considered a feminine aspect. And

we all can take physical action in the world like building a house, which would typically be considered a masculine aspect. Some qualities associated with the feminine are unconditional love, kindness, intuition, wisdom, and patience. I’d now like to ask you a few questions about how you have experienced the feminine nature in your life.

1. Can you please share about any experiences that you feel have **increased** or **expanded**

your feminine nature? Thank you for sharing.

1. How about any experience(s) that you feel have **reduced, diminished,** or **shut down** your feminine nature?
2. How would you describe your relationship with your mother or mother figure?
3. Is there anything else you would like to share about this topic of masculine and feminine natures?

*[If participant seems stressed in any way or the previous section was long and/or emotional…”Are you ok if we keep going, or would you like to take a break?”]*

1. Thank you for sharing about that. In this final section, I’d like to ask about your experience with specific emotions.
   - Have you ever experienced feelings of helplessness or hopelessness about something in your life?
     - If so, can you please share about that experience?

*[If they are not sure what these words mean...helplessness, which is a feeling like you were not able to defend yourself or to act effectively in your life or feelings of hopelessness, which is a feeling a sense of despair about something in your life]*

- - Have you ever experienced the emotion of intense anger or rage towards someone or something specific in your life, or about the world in general?
    - If yes, can you please share more about that?

Thank you so much for sharing about your emotions, I know these can be challenging to discuss and share openly. *[If participant seem stressed in any way…”Would you like to take a break, or shall we continue?”]*

We are almost done with the interview.

This last question is about posttraumatic growth.

1. **Posttraumatic growth** is defined as positive change experienced as a result of the struggle with a major life crisis or a traumatic event. We are all incredibly resilient and can be changed by encounters with life challenges, sometimes in radically positive ways. Are there any ways you feel your experience with breast cancer or other challenging events have positively changed or affected your life?

Is there anything else that you feel we should have asked or that you want to share about that you think we should know.

### Closing

Thank you so much for answering these questions openly and honestly. That was my final interview question and I’d like to ask you to take a moment and close your eyes. Take a few deep breaths and check in with yourself about how you are doing right now.

How are you feeling after the interview? Is there anything you would like to ask me?

We are very grateful for your support of the study. Please know that the results from this study will directly contribute to IONS programs in health and healing in the future.

### Logistics for getting paid:

I’m now going to move on to the logistics of your study compensation. You will be receiving your gift card(s) in the mail with a gift card receipt form and a self-addressed, stamped envelope. Please sign the gift card receipt and send it back to us as soon as you can.

### Follow-up Script:

*(Note to Interviewer: at the end of the call schedule a time in your calendar to call the person the next day and check in).*

The last thing I’d like to do today is let you know that I will be calling in about 24 hours or so to see if you have any questions or need support in any way. We know sometimes talking about these things can be challenging, so we wanted to check in with you. If I don’t happen to reach you, is it ok if I leave a VM?

If you are doing fine and don’t have any questions there is no need to reply. If you have any questions or need support please reach out to us with the study contact information.

Follow up script: “We talked about a lot yesterday, I'm just checking in to see if you feel ok and if you have any questions.”

(ONLY IF ASKED - 6-9 months, study will be published and you can get copy of results then)

- Check in about their contact with their mental health provider, if it seems necessary

*Provide a list of mental health support services if necessary.*

# Free Crisis Hotline Numbers

## [National Suicide Prevention Lifeline](https://suicidepreventionlifeline.org/talk-to-someone-now/): 1-800-273-TALK (8255)

This 24-hour hotline is available to anyone in crisis and provides free and confidential emotional support and crisis intervention.

## [Crisis Text Line](http://www.crisistextline.org/): Text “home” to 741741

This unique hotline is available via text message to anyone experiencing mental health difficulties or an emotional crisis. Highly trained counselors offer support and guidance to calm you down and make sure you are safe.

## [Substance Abuse and Mental Health Services Administration (SAMHSA) National](https://findtreatment.samhsa.gov/) [Helpline](https://findtreatment.samhsa.gov/): 1-800-662-HELP (4357)

SAMHSA’s helpline and web-based behavioral health treatment services locator can help you find information about treatment providers, therapists, counselors, support groups, and community resources in your area.

## [National Alliance on Mental Illness (NAMI) Helpline](https://www.nami.org/Find-Support/NAMI-HelpLine): 1-800-950-NAMI (6264)

The NAMI Helpline is available Monday through Friday, 10 a.m. to 6 p.m. EST to answer your general questions about mental health issues and treatment options.
